# THE RELATION AMONG THE PHYSICAL ACTIVITY LEVEL DURING LEISURE TIME, ANTHROPOMETRY, BODY COMPOSITION, AND PHYSICAL FITNESS OF WOMEN UNDERWENT OF BARIATRIC SURGERY AND AN EQUIVALENT GROUP WITH NO SURGERY

**DOI:** 10.1590/0102-6720201700040006

**Published:** 2017

**Authors:** Caio Machado de Oliveira TERRA, Caroline Ferraz SIMÕES, Anselmo Alexandre MENDES, Ronano Pereira OLIVEIRA, Rafaela Pilegi DADA, Victor Hugo de Souza MENDES, João Carlos LOCATELI, Nelson Nardo-Junior

**Affiliations:** 1 Physical Education Department, State University of Maringá, Maringá, PR;; 2 Post-graduate program UEM/UEL, State University of Maringá, Maringá, PR;; 3 Federal Tocantins Institute, Araguatins, TO;; 4 Educational Bureau, Marialva City Hall, Marialva, PR;; 5 Interdisciplinary Health Sciences Post-graduate `Program, Federal University of São Paulo, Santos, SP;; 6 Multidisciplinary Center of Obesity Studies, State University of Maringá, Maringá,PR), Brazil.

**Keywords:** Obesity, Bariatric surgery, Physical fitness and physical activity., Obesidade, Cirurgia bariátrica, Aptidão física e nível de atividade física.

## Abstract

***Background*:**

Bariatric surgery is an alternative to the obesity treatment.

***Aim*:**

To compare anthropometric variables such as body composition and physical fitness of those who performed Roux-en-Y gastric bypass.

***Methods*:**

Were evaluated 108 women. They were subdivided in three groups: those who performed the bariatric surgery by private health insurance (SAS, n=36); by the public health care (SUS, n=36), and an equivalent group which did not perform the surgery (NO, n=36). Were performed physical fitness, anthropometric and body composition tests. Was evaluated the level of physical activity during the leisure period.

***Results*:**

Statistically significant differences were observed between the groups sedentary operated (n=28) and sedentary non-operated (n=13) on anthropometry and fat percentage, being the highest indexes in the group operated.

***Conclusion*:**

The level of physical activity showed a positive influence related to anthropometric variables, body composition of the individuals who performed the bariatric surgery when compared to the ones non-operated.

## RESUMO

***Racional*:**

Observa-se o aumento da realização da cirurgia bariátrica como alternativa de tratamento para obesidade.

***Objetivo*:**

Comparar a antropometria, composição corporal e aptidão física de pacientes submetidos ao bypass gástrico em Y-de-Roux.

***Métodos*:**

Foram avaliadas 108 mulheres subdivididas em: operadas por convênios de saúde particulares (SAS, n=36), Sistema Único de Saúde (SUS, n=36) e um grupo equivalente não operado (NO, n=36). Foram realizados testes de aptidão física, antropometria e composição corporal, e avaliado nível de atividade física no lazer.

***Resultados*:**

Não houve diferença estatisticamente significativa nas variáveis estudadas entre os grupos SAS, SUS e NO. Observaram-se diferenças estatisticamente significativas entre os grupos operados sedentários (n=28) e não operados sedentários (n=13) na antropometria e percentual de gordura, sendo ao grupo operado os índices mais altos.

***Conclusões*:**

O nível de atividade física apresentou influência positiva sobre as variáveis antropométricas, de composição corporal de pacientes submetidos à cirurgia bariátrica quando comparados aos não operados.

## INTRODUCTION

The obesity is a multifactorial disease characterized by the fat excess in the organism^6^ ^,^ ^15^ ^,^ ^29^ ^,^ ^32^ The fat excess is also recognized as a pandemic, being a major problem of public health in both developed and developing countries. It is estimated that about 1,9 billion adults is already diagnosed with body mass excess worldwide, and out of this, 600 million are already obese^32^.

The growing number of obesity in its worst case (BMI≥40 kg/m^2^) is another concerning scenario^3^ ^,^ ^24^. In this way, the choice to treat obesity by the surgery method is more and more present nowadays, due to its cost effectiveness and short term benefits^3^ ^,^ ^7^ ^,^ ^20^ ^,^ ^30^. 

The bariatric is offer as high complexity assistance to people with severe obesity; however, this procedure is not total efficient and leads to a phase of adaptation, with behavior and habit changes in order to cause physical and psychological consequences if do not follow strictly^8^ ^,^ ^11^ ^,^ ^16^ ^,^ ^28^.

A common consequence very present after the performance of the bariatric surgery is related to decreasing of lean body mass^11^. This happens due to bad protein absorption and some intolerances acquired after the operation. The major part of the lean body mass consists of skeletal muscle mass and its lost reflects significantly on the decreasing of both static and dynamic muscle strength (physical fitness important aspects), and consequently decreasing the individuals functional capacity^11^ ^,^ ^19^ ^,^ ^28^.

Nevertheless, there are evidences that regular physical activity practice promotes an enhancement on the physical fitness parameters, and it contributes directly to the functional capacity and quality of life of the operated individuals^14^ ^,^ ^21^ ^,^ ^25^. Healthy lifestyle, including a healthy food intake and regular physical activity practice, contributes not only to the maintenance of the positive results acquired with the surgery, but also to the general health conditions of these patients^1^. 

Thus, the aim of this study was to compare the anthropometry, body composition, and physical fitness of patients underwent of bariatric surgery with non-operated individuals, dividing them in two distinct groups: active and sedentary.

## METHODS

Were assessed 320 women that presented the requirements of the pairing criteria by the body mass index (±2 kg/m^2^) and age (±10 years). Were excluded from the original sample 192 individuals that not filled the physical activity level questionnaire^18^. Therefore, the 108 women that were fit in both inclusion and exclusion criteria were divided into three groups. Two groups (SUS and SAS) represented the individuals operated with the Roux-en-Y gastric bypass, from 2000 to 2012, by the public health care (SUS) or particular insurances (SAS) respectively. A third non-operated equivalent control group (NO) underwent to the same assessments applied to other two groups.

The patients were invited to participate of the meetings, where was elucidated the research details, and asked to sign the free and informed consent previously approved by the local Ethics Committee (protocol 412/2008).

To the individuals included in the sample were performed anamneses to collect the following data: age, procedure date, and data related to the bariatric surgery. After that, was applied a physical activity level questionnaire, proposed by Larsson et al.^17^ as well anthropometric, body composition and physical fitness assessments.

### Anthropometric and body composition evaluations

The anthropometric and body composition measures were taken at the Multidisciplinary Center of Obesity Studies. The body mass was assessed by an multifrequency bioelectrical impedance analyzer (Octapolar, Inbody 520 model, Korea) with an scale attached and precision of 100 g, with maximal weight capacity of 250 kg. The patients were advised to follow rigorously the protocol proposed by Heyward^13^. The height was verified through a wall mounted stadiometer with precision of 0.1 mm. 

### Physical fitness evaluation

The patients underwent to a submaximal effort test, 6MWT (6 min walking test) used as an indicative of the cardiorespiratory fitness and functional capacity. The test started after a 5 min rest in the sitting position to the maintenance of the resting physiological responses. A hall was used to establish a 50 m distance, in which the individuals were instruct to go through the longest distance possible in 6 min. An experient instructor controlled the time, and the patients should stop completely after the buzzer, so the exact distance was recorded for each individual. Encouragement phrases were used each minute to make the individuals reach their best^2^ ^,^ ^27^.

### Flexibility

The test “sit and reach” was performed using the Wells chair to flexibility assessment. The patients were instructed to keep the soles of the feet in contact with the chair, with both arms and legs outstretched. The arms on the box surface with the hands folded with the top of the fingers united and hands turned down, making contact with the chair. They should reach the longest distance possible going through it slowly, with no bumps, standing in their longest distance for at least 2 s. The best of three attempts was recorded^9^.

### Grip strength test

A manual dynamometer was used (T-18, Smedley III) to perform the grip strength test through the following protocol: lateral legs stretching, arms in the orthostatic position, forearms and fists in a neutral position at the same line. The individual should make the maximal strength with their fingers to move the equipment bar. Three attempts were repeated and the best one was recorded^9^. 

### Statistical analysis 

To the descriptive and inferential data analysis was used the statistic package IBM, Software SPSS 20.0 version, and the normality data was tested and the significance level was established in ≤0.05). The data distribution was made by the Shapiro-Wilk test, and the following statistic tests: ANOVA One-Way, Kruskal-Wallis and Post-Hoc of Bonferroni were used the comparison between the three groups (SUS, SAS and NO). The t independent test and U of Man-Whitney compared the operated active and sedentary group, as well the Pearson’s correlation. Were considered in the data analysis only the patients that correctly answered the level of physical activity questionnaire. 

## RESULTS

The Table 1 presents the results in mean and standard deviation of the characterization variables. The tests did not showed any statistically significant difference in none of the variables analyzed, ensuring the groups homogeneity. 

**TABLE 1:** Sample characterization subdivided and comparison of anthropometric, body composition and physical fitness of the groups SAS, SUS and NO

Variables	SUS (n=36)		SAS (n=36)		NO (n=36)		p
Age (years)	50.0	(17.00)	49.5	(18.25)	44.0	(14.5)	.501
Body mass (kg)	72.45	(14.77)	78.45	(15.20)	71.75	(19.95)	.119
Height (m)	1.59	±0.07	1.59	±0.06	1.60	±0.07	.821
BMI (kg/m^2^)	26.93	(6.93)	30.64	(6.20)	28.25	(8.20)	.128
WC (cm)	82.65	(15.30)	84.00	(13.63)	85.75	(16.6)	.453
HC (cm)	103.0	(15.42)	111.65	(13.45)	105.25	(18.17)	.053
F%	36.39	±8.50	40.68	±6.78	39.1	±7.56	.060
SMM (kg)	23.95	(3.60)	24.45	(5.18)	24.45	(4.85)	.840
GS (kg/f)	27.00	6.30	25.63	±5.30	25.62	±4.68	.225
FLEX (cm)	24.50	15.75	20.14	±9.40	21.61	±6.69	.148
6MWT (m)	499.68	91.00	499.7	±65.92	521.4	±72.41	.297

*=Statistically significant difference; p=≤0,05; BMI=body mass index; WC=waist circumference; HQ=hip circumference; F%=fat percentage; SMM=skeletal muscle mass; GS=grip strength; FLEX=wells chair sit and reach test; 6MWT=6-minutes walking test; FLEX=flexibility; time since surgery SUS=38,0 (30); time since surgery SAS=61,0 (114,5). Anova one-away e Kruskal-Wallis

Was not observed any statistically significant difference between the SAS and SUS group regarding the physical fitness variables and their respective characterizations, being considered equivalent and subdivided according to the level of physical fitness during leisure time. Thus, were performed comparisons of the anthropometric, body composition, and physical fitness variables according to the sedentary group, comparison of the operated group (n=28) and NO (n=13) and between the physically actives operated (n=35) and NO (n=15). 

Was observed that, according to the Table 3, there are statistically significant differences between the sedentary groups (operated and non-operated) when analyzed the following variables: body mass, BMI, WC, HC, and F% (p=0,003, p=0,004, p=0,045, p=0,028, p=0,034 respectively). Of this variables, the highest indexes were found in the operated group, who presented median of the time since surgery of 46,0 (102).

**TABLE 2:** Correlation among anthropometric, body composition and health-related physical activity variables

	Mass	BMI	WC	HC	F%	SMM	GS	FLEX	6MWT
BM	1	.916***	.792***	.704***	. 678***	. 430**	. 260**	-.131*	-.213*
BMI		1	.831***	.670***	. 801***	. 293*	. 092*	-.094*	-.367**
WC			1	.515***	. 649***	. 238*	. 119*	-.167*	-.269*
HC				1	. 571***	. 243*	. 234*	. 013*	-.103*
F%					1	- .120*	- .110*	-.168**	-.445**
SMM						1	. 278*	-.101**	. 159*
GS							1	-.014*	. 365**
FLEX								1	. 017*
TC6M									1

*Weak correlation; **moderate correlation; ***Strong correlation; BM=body mass; BMI=body mass index; WC=waist circumference; HQ=hip circumference; F%=fat percentage; SMM=skeletal muscle mass; GS=grip strength; 6MWT=6 min walking test; FLEX=flexibility; n=72. Pearson.

**TABLE 3:** Comparison of the patients underwent to bariatric surgery and an equivalent non-operated group divided in physically active and sedentary during their leisure time

	Age	BM	BMI	WC	HC	F%	SMM	GS	FLEX	6MWT
Sed.										
Opera n=28	51.0 (18.0)	83.1 (12.9)	31.48 (17.1)	84.5 (15.0)	111.3 (18.1)	39.9 ±8.1	24.0 (4.1)	26.5 ±4.6	22.8 ±10.9	484.6 ±64.94
NO n=13	51.0 (19.0)	74.3 (17.8)	29.2 (7.3)	84.0 (32.5)	111 (11.5)	39.6 ±7.5	24.5 (5.1)	25.2 ±2.9	20.3 ±7.0	516.6 ±65.1
p	.923	.003*	.004*	.045*	.028*	.034*	.318	.937	.450	.053
Actives										
Opera n=35	45.5 (18.0)	70.75 (17.4)	27.0 (5.62)	82.65 (13.7)	103.4 (16.2)	36.8 ±7.4	24.5 (4.1)	26.9 ±5.6	22.2 ±9.27	509.23 ±58.91
NO n=15	51.0 (17.5)	76.1 (14.7)	31.3 (6.6)	84.25 (15.9)	111.5 (16.4)	39.87 ±7.83	24.4 (3.9)	26.2 ±4.29	22.17 ±10.1	492.6 ±65.6
p	.015*	.429	.675	.747	.675	.835	.116	.145	.297	.251

*=statistically significant difference; p<0,05; age=years; Sed.=sedentary; opera=operated; BM=body mass; BMI=body mass index; WC=waist circumference;HC=hip circumference; F%=fat percentage; SMM=skeletal muscle mass; GS=grip strength; 6MWT=6 min walking test; FLEX=flexibility. Age, body mass, BMI, WC, HC e SMM are presented in median and interquartile amplitude. Time since surgery for the physically active operated group=39,5 (42,0). Time since surgery for the sedentary operated group=46,0 (102,0). Performed tests=independent t student test and U of Mann-Whitney

## DISCUSSION

Studies previously demonstrated that there is a reduction on the skeletal muscle mass and bone mass through the years after the bariatric surgery. This fact may cause complications such as the decrease of the daily energy expenditure and impacts on the bone metabolism^5^ ^,^ ^8^ ^,^ ^31^, and consequently a decrease of the physical activity indexes; however, this last influence was not observed in the present study.

In a review study^10^ was verified the bariatric surgery is considered as a good alternative concerning the body mass loss; nevertheless, it should be aware to the individuals age group, although the induced weight loss has been shown itself as efficient increasing the lifetime of severe obese patients.

On Table 2 is observed the grip strength presents a weak positive correlation with SMM (r=0,159). In other hand, the 6MWT obtained a moderate negative correlation with F% (r=-0,445) and BMI (r=-367), and a weak negative correlation with C (r=0,269) and body mass (r=-0,213), demonstrating the relations between the body mass and the result obtained in the 6MWT. This data corroborate with another study with 43 obese that underwent to assessments both pre and post-late bariatric surgery, in which the results indicated that a high BMI negatively modulate the performance on the 6MWT^4^ ^,^ ^27^. In this sense, is possible to observe that in short-term, the bariatric surgery did not cause any negative impact on this variable. 

The Table 3 indicates that the operated and non-operated sedentary group presented statistically significant differences to the variables: body mass, body mass index, waist circumference, hip circumference, and F%. Regarding the both physically active operated and non-operated groups, was verified difference only on the variable age. Supporting these results, other studies made evident the relation between the regular practice of physical activity and an enhancement of the functional capacity and quality of life of operated patients^12^ ^,^ ^14^ ^,^ ^21^ ^,^ ^25^.

These results, corroborating with the ones in the scientific literature, indicate that Y-en-Roux by-pass with physical activities after the procedure, presents higher weight loss and enhance quality of life when compared to those considered inactive^17^. Santos et al. developed a prospective study in which were observed statistically significant differences in the adherence to the physical activity practice during the first 12 months post-operation. Although with sensible differences, it could be considered low frequency of physical practice regarding this specific population^22^.

The statistical comparison analysis between the operated active groups (n=35) and NO (n=15) did not demonstrate significant differences between the groups on the anthropometric, body composition, and physical fitness variables. In other study^28^ was demonstrated that patients with severe obesity (BMI 43 kg/m^2^), when underwent to a training post-surgery protocol, presented better maintenance and even increase in strength in comparison to their pairs who were not exposed to the same procedure. Was also observed in the same study a positive modulation of the aerobic and functional capacity assessed through the 6MWT.

The results on Table 3 confirm that the adoption of a healthy lifestyle, including the regular practice of physical activity contribute not only in the maintenance of the results acquired in the bariatric surgery^19^, but also enhance the general health conditions, and has an important role on the recuperation of the body mass lost during the first years after the surgery^18^.

In other hand, only the adoption of active habits during leisure time were seen as not enough to differ the groups regarding health-related physical fitness, functional capacity, and SMM. These parameters are very important to the age group of present study. However, the study demonstrated a positive impact on the anthropometric indexes between the operated sedentary group and NO, and of these parameters, BMI and WC - considered strong indicatives of cardiometabolic risk - being in the higher values observed in the sedentary operated group. 

Is important to emphasize that the absence of statistically significant differences presented on the operated and non-operated active groups may be related to the time after surgery in months in which the patients were (SAS=61,0 (114,5); SUS=38,0 (30,0) months). These periods are characterized as body mass and BMI acute recuperation phases.

The literature indicates the highest body mass loss occurs between de 12^th^ and 18^th^ months after the procedure^26^. However, the results lead to a gradual body mass gain until the 6^th^ year after the surgery, followed by a weight stabilization, leading to reach the same situation before the surgery until 8^th^ year. This way, is understandable that the body mass dependent variables may also follow this trend, explaining the similarity between the operated and non-operated groups, equivalent in the other variables. 

Were verified with the development of the present study that patients underwent to bariatric surgery that maintain physical activity health habits during the leisure period presents anthropometry, body composition, and physical fitness similar to a non-operated physically active group. However, the non-adoption of these health habits demonstrated a negative influence over the variables analyzed in the present study, because they present worse anthropometric and fat in comparison to those that did not performed the surgery. Thus, is emphasized the necessity of have health habits in order to keep the benefits acquired in the surgery procedure. 

## CONCLUSION

There are no differences between the patients underwent to Y-en-Roux gastric bypass by the public health care, particular health insurance and an equivalent non-operated group in comparison to anthropometric, body composition and physical fitness variables. Nevertheless, these variables demonstrated differences between themselves, indicating that better anthropometric and body composition parameters are correlated to better physical fitness conditions on the analyzed variables. 
